# Proteomic analysis of human plasma in chronic rheumatic mitral stenosis reveals proteins involved in the complement and coagulation cascade

**DOI:** 10.1186/1559-0275-11-35

**Published:** 2014-09-24

**Authors:** Somaditya Mukherjee, Mashanipalya G Jagadeeshaprasad, Tanima Banerjee, Sudip K Ghosh, Monodeep Biswas, Santanu Dutta, Mahesh J Kulkarni, Sanjib Pattari, Arun Bandyopadhyay

**Affiliations:** Cell Biology and Physiology Division, CSIR-Indian Institute of Chemical Biology, 4 Raja S. C. Mullick Road, Kolkata, 700032 India; Proteomics Facility, Division of Biochemical Sciences, CSIR-National Chemical Laboratory, Pune, 411008 India; General Medicine Department, Medical College, Kolkata, India; Department of Cardiology, Geisinger Community Medical Center & Wright Center for graduate medical education, Scranton, PA 18510 USA; Department of Cardio-thoracic and Vascular Surgery, Institute of Post Graduate Medical Education and Research, SSKM Hospital, Kolkata, 700020 India; Rabindranath Tagore International Institute of Cardiac Sciences, Kolkata, 700099 India

**Keywords:** Rheumatic fever, Mitral Stenosis, Plasma proteomics, Inflammation, Immunotechniques

## Abstract

**Background:**

Rheumatic fever in childhood is the most common cause of Mitral Stenosis in developing countries. The disease is characterized by damaged and deformed mitral valves predisposing them to scarring and narrowing (stenosis) that results in left atrial hypertrophy followed by heart failure. Presently, echocardiography is the main imaging technique used to diagnose Mitral Stenosis. Despite the high prevalence and increased morbidity, no biochemical indicators are available for prediction, diagnosis and management of the disease. Adopting a proteomic approach to study Rheumatic Mitral Stenosis may therefore throw some light in this direction. In our study, we undertook plasma proteomics of human subjects suffering from Rheumatic Mitral Stenosis (n = 6) and Control subjects (n = 6). Six plasma samples, three each from the control and patient groups were pooled and subjected to low abundance protein enrichment. Pooled plasma samples (crude and equalized) were then subjected to in-solution trypsin digestion separately. Digests were analyzed using nano LC-MS^E^. Data was acquired with the Protein Lynx Global Server v2.5.2 software and searches made against reviewed *Homo sapiens* database (UniProtKB) for protein identification. Label-free protein quantification was performed in crude plasma only.

**Results:**

A total of 130 proteins spanning 9–192 kDa were identified. Of these 83 proteins were common to both groups and 34 were differentially regulated. Functional annotation of overlapping and differential proteins revealed that more than 50% proteins are involved in inflammation and immune response. This was corroborated by findings from pathway analysis and histopathological studies on excised tissue sections of stenotic mitral valves. Verification of selected protein candidates by immunotechniques in crude plasma corroborated our findings from label-free protein quantification.

**Conclusions:**

We propose that this protein profile of blood plasma, or any of the individual proteins, could serve as a focal point for future mechanistic studies on Mitral Stenosis. In addition, some of the proteins associated with this disorder may be candidate biomarkers for disease diagnosis and prognosis. Our findings might help to enrich existing knowledge on the molecular mechanisms involved in Mitral Stenosis and improve the current diagnostic tools in the long run.

**Electronic supplementary material:**

The online version of this article (doi:10.1186/1559-0275-11-35) contains supplementary material, which is available to authorized users.

## Background

Acute rheumatic fever which is a sequelae of Group A Streptococcus throat infection in genetically susceptible individuals, remains the most common reason for Rheumatic Heart Disease (RHD). RHD is a chronic acquired heart disorder in children and young adults worldwide [1]. It is a major public health problem in Low and Middle Income Countries (LMICs) having a global prevalence of at least 15.6 million cases, with 282,000 new cases and registering 233,000 deaths each year [2]. In India, RHD accounts for 30-40% cardiovascular disease related hospital admissions [3].

RHD is a post-infection autoimmune disease. It is characterized by chronic inflammation of the myocardium and heart valves in particular. Mitral valve is most commonly affected followed by involvement of aortic valve. The tricuspid and pulmonary valves are usually less affected. RHD in mitral valve apparatus manifests most commonly as Mitral Stenosis or Mitral Regurgitation or combination of both producing hemodynamic burden on heart [4].

Mitral Stenosis refers to narrowing of the mitral valve resulting in obstruction of blood flow from the left atrium to the left ventricle. The mitral valve is damaged and deformed predisposing it to scarring and narrowing (stenosis) later in life. Rheumatic Mitral Stenosis is associated with thickening of the mitral valve leaflets and fusion of commissures and chordae tendineae together with fibrosis and calcification. Hypertrophy of the left atrium develops and may be followed by right sided heart failure and pulmonary edema. Echocardiography is the main imaging technique used to assess patients with Mitral Stenosis. Symptoms usually develop when the mitral valve area decreases below 2.5 cm^2^ and it is classified as “mild” stenosis. When the mitral valve area decreases below 1.5 cm^2^ it is regarded as “moderate” stenosis and when the area diminishes below 1.0 cm^2^ it is termed “severe” stenosis [5].

Echocardiography has an established role not only in diagnosis but also determines severity of Mitral Stenosis of rheumatic origin. Over the past 10 years, advances in the quantification of valvular stenosis have occurred. These newer techniques allow for the differentiation of mild from moderate and moderate from severe disease. However it is costly in countries with the highest disease burden and only available in limited tertiary health care facilities. In such regions, echocardiographic screening might be considered logistically unfeasible or secondary prophylaxis insufficiently developed to recommend screening. Moreover, being a highly operator/technician dependent imaging modality, there is a considerable chance of over or under-diagnosis of Rheumatic Mitral Stenosis with echocardiography alone. Technical pitfalls of image acquisition and echocardiographic machine settings must always be addressed as image quality can substantially affect the interpretation of images. A lack of adequate number of experts in echocardiographic diagnosis of Rheumatic Mitral Stenosis in endemic countries like India will prevent detection of subtle echocardiographic features as well. So the best strategy may be a combined echocardiographic and biomarker based approach to predict, diagnose and manage chronic Rheumatic Mitral Stenosis.

In recent years proteomics has emerged as a novel technology that has been extensively applied to cardiovascular disease research with the aim to understand the disease mechanisms. Adopting a proteomic approach to study the pathogenesis of Rheumatic Mitral Stenosis may throw some light on how the disease develops, since samples from various sources can be analyzed, like tissues, body fluids and circulating cells. In the present study we analyzed the proteome of plasma, a very useful clinical source for proteomic analysis in various pathologies. Plasma is an ideal biological sample due to its accessibility [6]. Moreover, as blood circulates through every organ and tissue of the body, it contains valuable information pertaining to the different physiological and pathological states of the organism [7]. The circulatory system thus provides a unique repertoire of proteins shed by cells and tissues that can potentially be used to assess both normal and disease conditions.

However, plasma is one of the most complex body fluids with a wide dynamic range of protein concentrations (9–10 orders). This presents an extreme analytical challenge in characterization of the plasma proteome as highly abundant proteins tend to mask low abundant ones [8]. This complexity exceeds the analytical capability of most proteomic approaches. For this reason, plasma analysis requires pre-fractionation. Saturation protein binding to a combinatorial peptide ligand library (CPLL) in combination with mass spectrometry has recently gained importance in analyzing the plasma proteome as an alternative approach to the more traditional and widely used method of “immunodepletion” [9–14].

To date, no proteomic study has investigated the difference between stenotic and non stenotic human plasma proteins in the context of Rheumatic Mitral Valve disease. In the present study, by adopting a label free proteomic approach we therefore analyzed the protein profile in control and patient plasma. Our results highlight that Rheumatic Mitral Stenosis is an active inflammatory process which manifests both inflammatory and thrombotic components. New information about these proteins will elaborate our knowledge of the physiology and etiology of this disease.

## Results

### Study design

**Figure 1:**
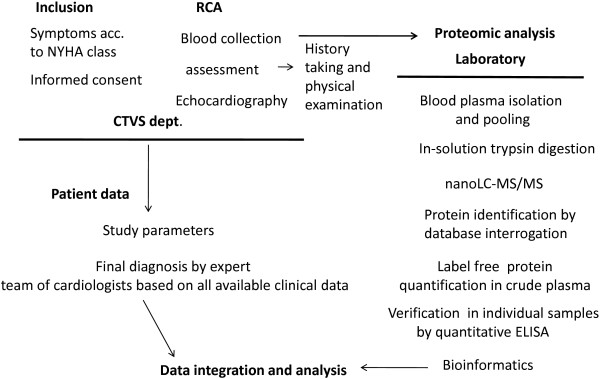
**Scheme depicting study design.** Pooled plasma samples isolated from peripheral venous blood of controls and patients are processed and analyzed as outlined in the chart.

Figure 1 presents an overview of the proteomic workflow adopted in the study.

### Baseline characteristics

Baseline characteristics of control and patient subjects are shown in Table 1. Diastolic blood pressure, left atrial (LA) diameter and left ventricular ejection fraction were found to be significantly different in the patient group than the control group. In fact, left atrial enlargement as understood from the LA diameter is most common in patients with chronic Rheumatic Mitral Stenosis. Patients and control subjects were age and sex matched. As shown in Table 2, 66% of the patients recruited in the study had history of rheumatic fever in childhood and 34% had atrial fibrillation. 46% of the patients belonged to New York Heart Association (NYHA) class I, 31% belonged to class II, 17% in class III and the remaining 6% in class IV. Drugs administered to patients in the study are shown (Table 3). Around 2% of the patients were taking diuretics, 71% digoxin, 31% aldactone, around 6% ACE inhibitors and 20% were taking beta blockers. Almost 51% of the patients were under anticoagulant (warfarin) therapy. All patients included in the study were under rigorous penicillin prophylaxis to prevent recurrent episodes of Acute Rheumatic Fever.

**Table 1:** **Baseline Characteristics of Controls and Mitral Stenosis patients**

Variables	Control (n = 19)	Rheumatic MS (n = 35)	p value
Age (years)	32.68 ± 1.77 (n = 19)	36.74 ± 1.43 (n = 35)	0.11
Male / Female	12 / 7	20 / 15	0.78
Clinical Presentation data			
Pulse (bpm)	76 ± 1.82	78.37 ± 2.77 (n = 35)	0.96
SBP (mmHg)	119.79 ± 3.03	114.86 ± 2.60 (n = 35)	0.29
DBP (mmHg)	82.26 ± 1.42	75.60 ± 2.15 (n = 35)	0.03
**Echocardiographic parameters (Normal range)**			
LA (20 – 40 mm)	30.88 ± 0.85 (n = 17)	48.58 ± 1.44 (n = 32)	<0.0001
LVIDd (35–56 mm)	43.53 ± 1.12 (n = 17)	44.77 ± 1.23 (n = 35)	0.52
LVIDs (24 - 42 mm)	29.24 ± 0.79 (n =17)	30.99 ± 0.93 (n = 35)	0.23
LVPW (6 -11 mm)	8.35 ± 0.26 (n = 17)	8.63 ± 0.26 (n = 32)	0.32
IVSD (6–11 mm)	8.53 ± 0.23 (n = 17)	9.11 ± 0.26 (n = 33)	0.12
EF (%)	66.82 ± 0.83 (n = 17)	56.80 ± 1.16 (n = 35)	<0.0001
PASP (mmHg)	-	57.32 ± 4.22 (n = 22)	NA
MVA (sq.mm)	-	87 ± 4 (n = 33)	NA
Fractional shortening (%)	32.73 ± 1.04 (n = 17)	30.71 ± 0.94 (n = 35)	0.19

*bpm* beats per minute, *EF* ejection fraction, *IVSD* interventricular septal diameter, *LA* left atrium, *LVIDd* left ventricular internal diameter diastolic, *LVIDs* left ventricular internal diameter systolic, *LVPW* left ventricular posterior wall, *MVA* mitral valve area, *n* number of subjects, *NA* not applicable, *NYHA* New York Heart Association, *PASP* pulmonary artery systolic pressure, *SBP* systolic blood pressure. Values indicate mean ± standard error of mean. p < 0.05 considered statistically significant between two groups.

**Table 2:** **Clinical Characteristics of patients with Rheumatic Mitral Stenosis**

Patient	Age	Sex	h/o RF	NYHA	Pulse	AF	SBP	DBP	Type of MS
*P1*	*41*	*M*	*Y*	*I*	*80*	*N*	*120*	*70*	*Severe MS + Moderate AR*
P2	42	F	N	II	88	Y	110	80	severe MS + Moderate MR + mild AR
P3	45	M	Y	I	72	N	130	90	Severe MS + Moderate MR + Mild AS + Moderate TR
P4	35	M	Y	I	60	N	110	60	Severe MS + Moderate AR + Moderate TR + Mild MR
P5	24	M	N	I	78	Y	110	66	Severe MS + Moderate MR
P6	26	M	N	I	78	N	120	90	Moderate MS + Mild AR
*P7*	*33*	*F*	*Y*	*I*	*78*	*N*	*130*	*90*	*Severe MS + Mild AR*
P8	35	M	Y	I	60	Y	120	90	Severe MS + Moderate TR + Mild MR
*P9*	*45*	*F*	*Y*	*III*	*77*	*N*	*100*	*70*	*Severe MS*
P10	35	M	N	III	80	N	100	60	Moderate MS
P11	33	M	N	I	66	N	100	80	Severe MS + Mild MR + Mild TR + Mild AS
P12	36	F	Y	IV	84	N	110	60	Severe MS + Severe TR
P13	48	M	Y	I	84	N	120	80	Severe MS
P14	25	F	Y	I	70	Y	100	60	Severe MS
P15	35	F	N	I	96	N	100	70	Severe MS + Mild TR
P16	32	F	N	I	58	Y	80	50	Severe MS
P17	55	M	N	II	60	Y	140	80	Severe MS
P18	33	F	Y	I	76	N	100	70	Severe MS + Mild MR
*P19*	*40*	*F*	*N*	*I*	*108*	*N*	*110*	*80*	*Severe MS*
P20	19	M	N	I	70	N	100	70	Severe MS
P21	38	F	Y	I	64	Y	80	50	Severe MS + Mild MR + Moderate AR
*P22*	*42*	*M*	*Y*	*III*	*64*	*N*	*140*	*100*	*Severe MS + Moderate AS + Moderate AR*
P23	24	F	Y	III	120	N	110	80	Severe MS + Moderate AS
*P24*	*27*	*M*	*Y*	*II*	*62*	*Y*	*130*	*100*	*Severe MS + Mild MR*
P25	48	M	Y	II	100	N	140	100	Severe MS + Moderate TR
P26	40	F	Y	III	80	N	110	70	Severe MS + Mild MR
P27	28	M	Y	II	70	N	110	70	Severe MS + Moderate MR
P28	35	F	Y	II	80	Y	120	80	Severe MS + Moderate TR + Mild MR
P29	43	F	Y	II	100	Y	120	80	Severe MS + Severe TR + Moderate AR
P30	35	M	Y	II	100	Y	120	70	Severe MS + Moderate AR
P31	50	M	Y	II	80	N	130	70	Severe MS + Moderate AS + Moderate AR
P32	47	F	Y	II	110	N	130	80	Severe MS + Moderate MR + Moderate TR + Moderate AR
P33	46	M	Y	IV	50	Y	140	80	Severe MS + Mild MR + Severe TR
P34	30	M	N	III	60	N	120	80	Moderate MS + Mild AR
P35	36	M	N	II	80	N	110	70	Severe MS + Mild AR + Severe TR

*AF* atrial fibrillation, *AR* aortic regurgitation, *AS* aortic stenosis, *bpm* beats per minute, *DBP* diastolic blood pressure, *F* female, *h/o RF* history of Rheumatic Fever, *M* male, *MR* mitral regurgitation, *MS* mitral stenosis, *N* no, *NYHA* new york heart association, *P* patient, *SBP* systolic blood pressure, *TR* tricuspid regurgitation.

**Table 3:** **Medications taken by Mitral Stenosis patients during the study**

Number	Diuretic	Lanoxin	Aldactone	ACE inhibitor	Beta blocker	Warfarin
***P1.***	*Y*	*Y*	*Y*			
**P2.**	Y	Y	Y			Y
**P3.**	Y	Y			Y	
**P4.**	Y		Y		Y	
**P5.**	Y	Y				Y
**P6.**	Y	Y		Y		
***P7.***	*Y*	*Y*				
**P8.**	Y	Y				Y
***P9.***	*Y*	*Y*	*Y*			*Y*
**P10.**	Y	Y	Y		Y	
**P11.**						
**P12.**	Y					
**P13.**	Y	Y	Y		Y	Y
**P14.**	Y	Y				
**P15.**						Y
**P16.**	Y	Y		Y		
**P17.**						Y
**P18.**	Y	Y	Y			Y
***P19.***	*Y*	*Y*				
**P20.**	Y	Y	Y			Y
**P21.**						Y
***P22.***	*Y*	*Y*				*Y*
**P23.**	Y		Y			
***P24.***	*Y*	*Y*				
**P25.**		Y				
**P26.**						Y
**P27.**	Y	Y				
**P28.**	Y	Y				
**P29.**	Y	Y				Y
**P30.**		Y	Y			Y
**P31.**	Y				Y	Y
**P32.**	Y	Y				Y
**P33.**	Y	Y			Y	
**P34.**					Y	Y
**P35.**		Y	Y			Y

*ACE* inhibitor Angiotensin Converting Enzyme inhibitor, *P* Patient, *Y* Yes.

### Proteomics findings

**Figure 2:**
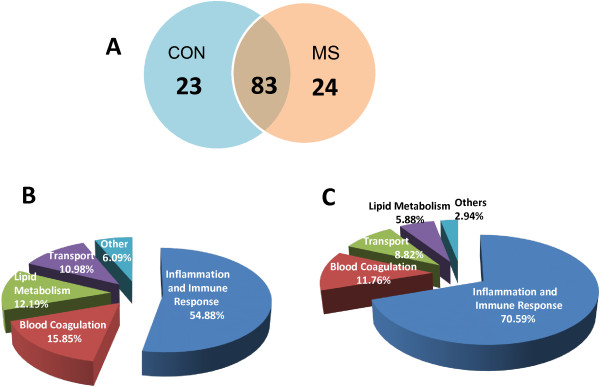
**Proteomics findings. (A)** Venn diagram comparing the number of identified proteins for the six biological replicates and their relationship. The numbers of proteins detected with at least two peptides are indicated by numbers. **(B)** Pie chart representing the distribution of identified proteins in the common or overlapping group according to their biological function. **(C)** Pie chart representing the distribution of identified proteins in the differentially altered group according to their biological function. Assignments are made on the basis of information provided by Swiss-Prot.

For analyzing crude and pre-fractionated plasma, stenotic and non-stenotic plasma samples were selected for proteomic analysis by nanoLC-MS^E^. This led to the identification of 130 proteins covering a range of molecular masses spanning 9–192 kDa and a pI range covering 4.4-9.8 (see Additional file 1 and Additional file 2). All these proteins were identified in at least two of the three technical replicates with two or more peptides matched. A total of 106 proteins were identified in pooled samples of the control group and 107 proteins in pooled samples of the patient group respectively. Of these, 83 were common to both control and patient groups (Figure 2A). However 23 proteins were unique to the control group and 24 proteins to the patient group.

Among the proteins identified in this comparative proteomics study, 34 proteins were found to be differentially abundant. Immunoglobulin heavy chains like α, γ and light chains κ and λ were significantly downregulated (i.e.; abundance of these protein species were higher in controls than patients) in Mitral Stenosis group. Complement proteins like C3, C4A, isoform 2 of complement C4A, C4B, complement C4b binding protein α chain, complement factor B, complement factor H were also found to be significantly downregulated in the patient group. Other proteins like α-1-acid glycoprotein I, α-2-macroglobulin, α-2-HS-glycoprotein, ceruloplasmin, α-1-antitrypsin, α-1-antichymotrypsin, haptoglobin, haptoglobin related protein were all downregulated. Among the apolipoproteins, Apo AI and Apo CIII were significantly downregulated (2.1-fold and 2.5-fold respectively) in patients. Also, isoform 2 of fibrinogen α chain, fibrinogen β chain as well as isoform γA of fibrinogen γ chain were significantly downregulated by 1.9-fold, 2.5-fold and 2.7- fold respectively in the patient group (Table 4).

**Table 4:** **Differentially abundant proteins found in the proteomic analysis**

Swiss-prot accession number	Protein name	Ratio [Patient/Control]	p value
P02763	Alpha 1 acid glycoprotein I	0.15	<0.05
P01011	Alpha 1 antichymotrypsin	0.60	0.01
P01009	Alpha 1 antitrypsin	0.32	<0.05
P02765	Alpha 2 HS glycoprotein	0.71	0.02
P01023	Alpha 2 macroglobulin	0.56	<0.05
P02647	Apolipoprotein A I	0.47	<0.05
P02656	Apolipoprotein C III	0.39	0.02
P04003	C4b binding protein alpha chain	0.29	<0.05
P00450	Ceruloplasmin	0.66	<0.05
P01024	Complement C3	0.67	<0.05
P0C0L4	Complement C4 A	0.64	<0.05
P0C0L5	Complement C4 B	0.69	<0.05
P00751	Complement factor B	0.46	<0.05
P08603	Complement factor H	0.41	<0.05
P02675	Fibrinogen beta chain	0.39	<0.05
P00738	Haptoglobin	0.21	<0.05
P00739	Haptoglobin related protein	0.33	<0.05
P01876	Ig alpha 1 chain C region	0.23	<0.05
P01877	Ig alpha 2 chain C region	0.61	<0.05
P01857	Ig gamma 1 chain C region	0.87	<0.05
P01859	Ig gamma 2 chain C region	0.38	<0.05
P01860	Ig gamma 3 chain C region	0.06	<0.05
P01861	Ig gamma 4 chain C region	0.84	<0.05
P01777	Ig heavy chain V III region TEI	0.60	0.02
P01834	Ig kappa chain C region	0.38	<0.05
P01620	Ig kappa chain V III region SIE	0.46	<0.05
P0CG04	Ig lambda 1 chain C regions	0.09	<0.05
P0CG05	Ig lambda 2 chain C regions	0.54	<0.05
P0CG06	Ig lambda 3 chain C regions	0.10	<0.05
P01871	Ig mu chain C region	0.42	<0.05
P0C0L4-2	Isoform 2 of Complement C4 A	0.64	<0.05
P02671-2	Isoform 2 of Fibrinogen alpha chain	0.53	<0.05
P02679-2	Isoform Gamma A of Fibrinogen gamma chain	0.37	<0.05
P02768	Serum albumin	0.83	0.0004

Three nano LC-MS^E^ analyses of the plasma samples were performed for protein identification and relative quantification.

### Functional annotation of profiled proteins

The 83 proteins common to both the groups and the 34 proteins found to be differentially altered were classified by functional annotation. In both cases, the majority of proteins could be classified into 4 different functional categories directly associated with important biological processes in the cardiovascular system: Inflammation and Immune response; Blood homeostasis and coagulation; Lipid Metabolism; Transport and Others (Figure 2B and 2C). These functional categories were mainly determined using the Swiss-Prot database. The proportion of inflammation related proteins increased from about 55% in the overlapping group to about 70% in the differentially regulated group.

### Network connections of proteins for further investigations

**Figure 3:**
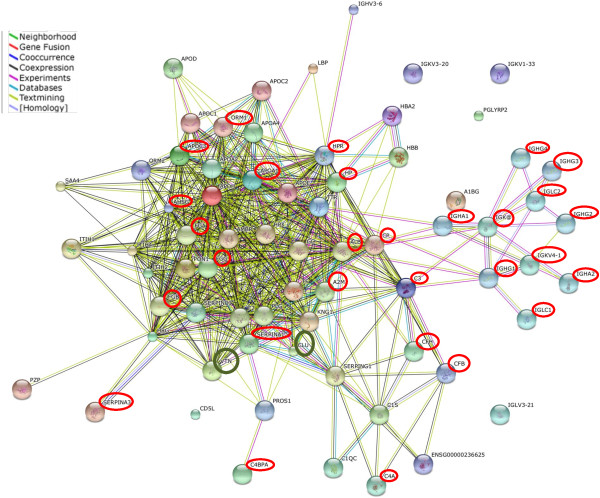
**Biological network of reported proteins associated with Rheumatic Mitral Stenosis.** Proteins marked by red circles indicate differentially abundant proteins. Proteins indicated by green circles have not been found to be differentially abundant in the proteomic phase but were found to be so in the verification phase of the study. AHSG, α-2-HS- glycoprotein; A2M,α-2-macroglobulin; ALB,albumin; APOA1,Apolipoprotein AI; APOC3,apolipoprotein CIII; CP,ceruloplasmin; CLU,clusterin; C3,complement component 3; C4A,complement component 4A; C4BPA,complement component 4 binding protein α; CFB,complement factor B; CFH,complement factor H; FGA,fibrinogen α chain; FGB,fibrinogen β chain; FGG,fibrinogen γ chain; HP,haptoglobin; HPR,haptoglobin related protein; IGHA1,Ig alpha-1 chain C region; IGHA2,Ig alpha-2 chain C region; IGHG1,Ig gamma-1chain C region (G1m marker); IGHG2,Ig gamma-2 chain C region (G2m marker); IGHG3,Ig gamma-3 chain C region (G3m marker); IGHG4,Ig gamma-4 chain C region (G4m marker); IGK@,Ig kappa chain C region; IGKV4-1,Ig kappa chain variable 4–1; IGLC1,Ig lambda-1chain C region; IGLC2,Ig lambda-2 chain C region; ORM1,orosomucoid 1 or α-1-acid glycoprotein 1; SERPIN A1,serpin peptidase inhibitor, clade A member 1 or α-1-antitrypsin; SERPIN A3,serpin peptidase inhibitor, clade A member 3 or α-1-antichymotrypsin; VTN,vitronectin

As seen in Figure 3, differential proteins like complement factor B, complement factor H, C4b binding protein α chain, orosomucoid I or α-1-acid glycoprotein I, α-1-antitrypsin, α-2-macroglobulin, haptoglobin, ceruloplasmin, albumin, apolipoproteins AI and CIII together with the fibrinogens were highly networked. Another set of proteins forming a novel module of network included the various immunoglobulin variants. These kinds of networks suggest the collaborative interactions between several protein candidates; in this case, of common proteins and/or differential proteins.

### Quantification of selected marker candidates in crude plasma samples by colorimetric ELISA

**Figure 4:**
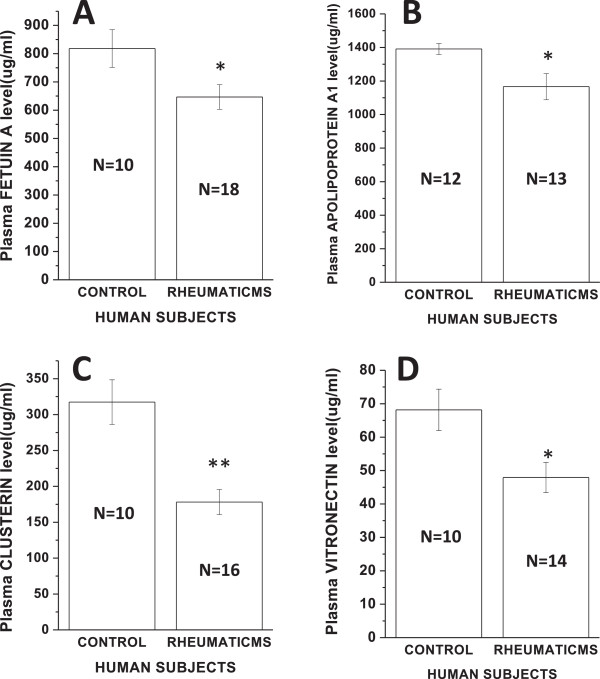
**Verification of selected protein candidates in normal and Mitral Stenosis subjects.** Plasma concentration of **(A)** α-2-HS-glycoprotein or fetuin A, **(B)** apolipoprotein AI, **(C)** clusterin and **(D)** vitronectin. Statistical significance is determined by unpaired, two-tailed Student’s *t*-test (*signifies p < 0.05 vs. control and **signifies p < 0.01 vs. control). Data represent mean ± SEM.

To assess the performance of the potential marker candidates identified by nano LC-MS^E^ analysis, their abundance in crude plasma of individual control and patient subjects were determined by immunoassay (ELISA or immunoturbidimetry). Several criteria were used to select potential candidates for ELISA validation. The protein candidates had to fulfill the following: (1) identification of the selected protein candidates in triplicate runs in both crude and equalized pooled plasma of patient and healthy subjects; (2) difference in levels should be statistically significant i.e., p < 0.05 and consistent regulation in all the replicate analyses; (3) potential biological relevance of these proteins to heart valve and/or cardiovascular disease based on information obtained from functional annotation of identified proteins and manual literature search of publications and lastly; (4) the availability of commercially available ELISA kits or primary antibodies. As half of the patients were on anticoagulant therapy, many of the candidate markers of coagulation or some of the protease inhibitors known to be involved in the coagulation cascade, could not be validated as this could have led to erroneous estimation of their circulating levels under the disease condition. Plasma α-2- HS-glycoprotein concentration was significantly lower in stenotic subjects than controls (818.3 ± 66.8 vs. 646.8 ± 43.5 μg/ml, p = 0.03; unpaired *t*-test; Figure 4A). Plasma level of apolipoprotein AI was also significantly lower in patients with Mitral Stenosis than healthy control subjects (1390.8 ± 33.1 vs.1166.2 ± 78.2 μg/ml, p = 0.02; unpaired *t*-test; Figure 4B). From the network map of the common and differential proteins in Figure 3, it is seen that apolipoprotein AI is predicted to interact with proteins like vitronectin and clusterin which have also been identified in control and patient plasma in this study. Hence, to check their status in chronic Mitral Stenosis, circulating levels of these proteins were also measured by quantitative ELISA. Plasma clusterin levels were significantly lower in patients than control subjects (317.4 ± 31.1vs.178.2 ± 17.5 μg/ml, p < 0.0001; unpaired *t*-test; Figure 4C). Lastly, plasma level of vitronectin was also significantly lower in patients with Mitral Stenosis than control subjects (68.2 ± 6.2 vs. 47.9 ± 4.5 μg/ml, p = 0.01; Figure 4D).

### Identification of pathways associated with Rheumatic Mitral Stenosis

**Figure 5:**
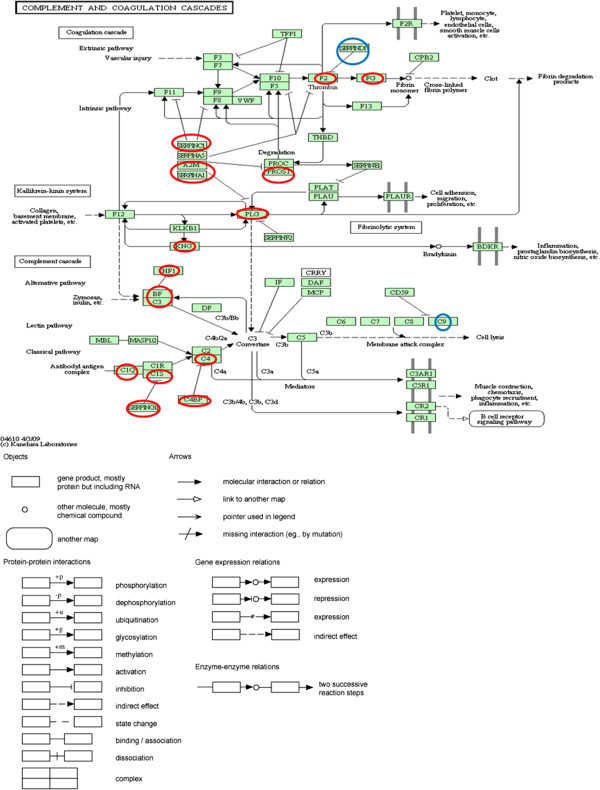
**Identification of pathways associated with Rheumatic Mitral Stenosis.** Proteins marked by red circles indicate those that are found to be differentially abundant and/or common to the control and patient groups. Proteins indicated by blue circles are identified only in the patient group. A2M, α-2-macroglobulin; BF, complement factor B; C1Q, complement component 1,q subcomponent; C1S, complement component 1, s subcomponent; C3,complement component 3; C4,complement component C4; C4BP, complement component 4 binding protein; C9, complement component C9; F2,coagulation factor II or prothrombin; FG, fibrinogen; HF1,complement factor H; KNG, kininogen 1; PLG, plasminogen; PROS1,protein S α; SERPIN A1,serpin peptidase inhibitor, clade A member 1 or α-1-antitrypsin; SERPINC1,serpin peptidase inhibitor, clade C, member 1 or antithrombin III; SERPIND1,serpin peptidase inhibitor, clade D, member 1or heparin cofactor II; SERPING1,serpin peptidase inhibitor, clade G, member 1 or C1 inhibitor.

Pathway analysis for the common and differentially abundant proteins in Rheumatic Mitral Stenosis revealed that about 18 of them belong to the complement and coagulation cascade (Figure 5). This is relevant in the context of the present study since chronic Rheumatic Mitral Stenosis represents an ongoing inflammatory state.

### Histopathology analysis

**Figure 6:**
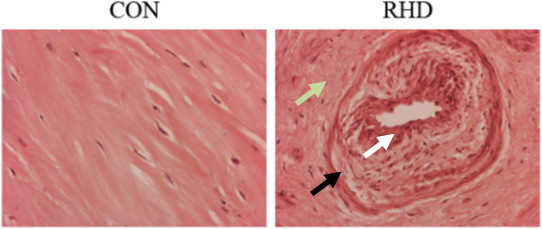
**Histopathological analysis.** Representative images (100 X magnification) of hematoxylin-eosin stained sections of 1 normal mitral valve (control) and 3 stenotic mitral valve samples (RHD). Rheumatic mitral valve tissue section shows abundance of inflammatory cells (white arrow head), fibrosis (green arrow) and neovascularization (black arrow). Normal mitral valve section is devoid of inflammatory cells and neovascularization (black arrow).

Microscopic examination of hematoxylin - eosin (HE) stained mitral valve sections from Rheumatic Mitral Stenosis subjects showed enhanced degree of fibrosis and neovascularization (arrows marked in Figure 6). Focal perivascular mild infiltration of lymphocytes and abundant plasma cells were prominent, thus confirming inflammatory aetiology in those subjects, consistent with the findings from pathway analysis as well as from functional annotation of profiled proteins. In contrast, normal mitral valve was composed of loose collagen tissue without blood vessels and inflammatory cells (Figure 6).

## Discussion

The aim of the present study was to provide a better understanding of the pathophysiological mechanisms involved in Rheumatic Mitral Stenosis. To accomplish this, we performed two distinct analyses - using crude plasma and a different strategy to reduce the complexity of the plasma proteome (equalization). To our knowledge, most of the proteins identified in this study have not been previously described in the context of Rheumatic Mitral Stenosis.

To increase the detectable proteome dynamic range we used an alternative pre-fractionation method, CPLL - a technology designed for sample equalization by using a combinatorial library of hexapeptides bound to a chromatographic support. Plasma equalization reveals proteins that are not detected in crude plasma samples like serum amyloid A4, lipopolysaccharide binding protein, vitamin K dependent protein S, paraoxonase 1 and so on. Although plasma equalization combined with the power of nano LC-MS^E^ helps to uncover less abundant proteins like ficolin 3 (21.6 μg/ml) [15], sex hormone binding globulin (8.1 ng/ml) [16] and angiotensinogen (1.5 μg/ml) [17], the depth of proteome coverage is low. However, many of the abundant proteins particularly much of the immunoglobulins, α-1-acid glycoprotein, α-2-macroglobulin, haptoglobin and serotransferrin are lost in this approach.

To further understand the significance of a plethora of proteins that may appear in the plasma in response to diverse physiological events in Mitral Stenosis, we compared the protein abundance patterns in crude plasma between Rheumatic Mitral Stenosis patients and normal controls using label-free proteomic technique. We identified 34 differential proteins which are downregulated in the plasma of patients. Proteins that are differentially abundant between the two groups are potential serological biomarkers for Mitral Stenosis. In addition, proteins that are differentially abundant in patients could reflect pathophysiological changes arising from abnormal mitral valve structure and function. These proteins may be used for diagnosis and disease monitoring.

To gain insight into the biological significance of the altered and the common proteins in the disease, the differentially abundant and overlapping proteins are categorized according to their reported biological functions. Functional characterization of these proteins of interest reveal that more than 50% are involved in inflammation and immune response. This is corroborated by findings from pathway analysis as well as from histopathological studies on excised tissue sections of stenotic mitral valves. Interestingly, evidences of chronic inflammation with cellular infiltration have been previously reported by several authors in aortic stenotic valves [18, 19].

Most of the differences are found in proteins related with Inflammation and Immune response in the present study. Alterations in the complement system proteins particularly downregulation of C4A and its isoform, C4B, complement factor H, complement factor B, C3 and C4b binding protein α chain along with several forms of immunoglobulins are observed in plasma of Mitral Stenosis patients in the label free protein quantification study. The complement system is the most important component of humoral autoimmunity in the natural defense mechanism in man. The system is not only vital for host defense against infectious diseases, it also plays an important role in protection, development, and progression of inflammatory diseases including many rheumatic disorders [20]. The presence of complement C1 proteins and C3 molecule clearly shows that both the 'classical’ and 'alternate’ pathways are operational in the disease process. Complement C4, an essential component of the humoral immune response, plays a central role in the activation of the classical and lectin pathways of the complement system. Chronic infections can initiate formation of immune complexes, causing continuous activation and consumption of complement [21]. This may be the reason for the lower abundances of C3, C4A, C4B and C4b binding protein α chain observed in Mitral Stenosis patients. In fact, low C4b copy number carrier status has been reported in patients with acute myocardial infarction and stroke [22]. The identification of proteins such as serum amyloid P component, which has been shown to bind some complement components ficolin 3 (collagen/fibrinogen domain containing lectin 3p35) and galectin 3 binding protein (basement membrane autoantigen p105 or lectin galactoside binding soluble 3 binding protein) in patients together point to the fact that possibly the lectin pathway of complement also plays a role in the pathophysiology of Rheumatic Mitral Stenosis. All these data suggest that there is a strong innate response in Mitral Stenosis. Thus it is an active inflammatory process that could lead to a prothrombotic state.

It is known that protease inhibitors and proteases play crucial roles in various physiologic functions as blood homeostasis and coagulation, transport, extracellular matrix turnover and tissue remodelling as well as triggering or modulating inflammatory processes in pathologic situations. Some of these proteins (α-1-antichymotrypsin, α-1-antitrypsin, inter α trypsin inhibitors, protein AMBP) had previously been implicated in atherosclerosis, aortic valve stenosis and other cardiovascular diseases [23–25]. Our analysis detects this group of proteins in Mitral Stenosis patients. Serine protease inhibitors (SERPINs) represent a major regulatory mechanism controlling enzyme activity of activated coagulation factors. Under normal blood circulation conditions, these proteins are present in sufficient concentrations to further down-regulate the various clotting factors thereby acting as security against random activation of thrombin or other proteases and the resulting fibrin clots. Serpins such as antithrombin III (SERPIN C1) and heparin cofactor II (SERPIN D1) have been detected in the present study. Both the proteins possess anticoagulant properties as they tightly bind and inactivate thrombin.

We also show a downregulation of circulating anti-proteases like α-1-antichymotrypsin (SERPIN A3) and α-1-antitrypsin (SERPIN A1) perhaps pointing to the development of a more prothrombotic state in Rheumatic Mitral Stenosis. This pro-coagulant effect may limit infection by trapping pathogens in local blood clots. A disbalance in blood homeostasis and coagulation system, leading to a local prothrombotic state of the aortic valve, has been previously reported [26]. We demonstrate a reduced abundance of proteins involved in blood homeostasis and coagulation particularly fibrinogen. Prothrombin is a serine protease that is synthesized in the liver and contains 10 Gla residues in its amino-terminal domain. Factor Xa cleaves prothrombin into its activated form thrombin. Thrombin in turn acts as a serine protease that converts soluble fibrinogen into insoluble strands of fibrin, as well as catalyzing many other coagulation-related reactions. Endogenous coagulation inhibitors like vitamin K dependent protein S, a cofactor of protein C are also detected in this study. Protein C is a Gla containing protease which when activated can proteolytically cleave Factors Va and VIIIa together with its cofactor protein S. Proteolysis can, therefore, be a method of regulating biological processes by turning inactive proteins into active ones.

In addition to abundance, post-translational modifications also have illustrated roles in governing activities of certain blood clotting factors. Synthesis of a unique modified glutamate residue called carboxyglutamate (Gla) results from post-translational modifications of newly synthesized inactive coagulation factors like Prothrombin in the liver endoplasmic reticulum by a vitamin K-dependent carboxylase [27]. This is termed as γ carboxylation which is an irreversible posttranslational modification that plays a role in the effective functioning of blood coagulation factors. In gamma-carboxylation, a carboxyl group is attached to certain Glu side chains of the protein resulting in the conversion of Glu to γ –carboxyglutamate (Gla). Phosphorylation of prothrombin has also been observed. Hence, our data in this study indicates an important deregulation or impaired function of proteases, anti-proteases and coagulation proteins in Mitral Stenosis development.

In our analysis, we detected several apolipoproteins (Apo AI, Apo AII, Apo AIV, Apo CI, Apo CII, Apo CIII, Apo D, Apo E) in control and patient plasma. In addition, we found alterations in the abundances of apolipoproteins AI and CIII in the “proteomic” phase of the study. Apolipoprotein AI is one of the dominant proteins in high density lipoprotein (HDL) but the other apolipoproteins identified in the present study have also been described as HDL components [28]. Recently a proteomic study has reported that HDL carries protein families implicated in complement activation, regulation of proteolysis, and acute-phase response [29]. It is further known that under inflammatory conditions anti inflammatory proteins such as paraoxonase 1 and clusterin are displaced from HDL by proinflammatory ones like serum amyloid A and haptoglobin. Thus anti-inflammatory HDL turns proinflammatory which is also called dysfunctional HDL [30]. All these signify that HDL may serve as a link between lipid metabolism, inflammation and mitral valve disorder of rheumatic etiology. This is an interesting aspect of this disease that has not been studied much so far but will be the pursuit of future investigations. It is believed that oxidation of low density lipoprotein in blood leads to heart ailments. The presence of glutathione peroxidase 3 in equalized plasma of Rheumatic Mitral Stenosis raises the possibility of a protective role in this pathology, since it modulates oxidative stress by increasing resistance of cells to such kind of vascular injury. Structural proteins like fibulin 1 and gelsolin together with its isoform in Rheumatic Mitral Stenosis point to the fact that possibly cells undergo structural remodelling both in terms of cytoskeletal elements and extracellular matrix. This group of proteins is totally non-existent in the control group. However further in-depth studies are warranted to substantiate these preliminary observations.

We focused on choosing common and differential protein candidates to develop a predictive network model for further biological investigation using Search Tool for the Retrieval of Interacting Genes/Proteins (STRING) database. This was done using data from biological functions of local networks surrounding the protein candidates [31–34]. In addition, to verify the findings from the pilot phase we have tested the abundance of a panel of four candidate serum proteins (α-2-HS-glycoprotein, apolipoprotein AI, clusterin and vitronectin) by immunoassays. The results confirm that the mean plasma level of these candidate proteins in Mitral Stenosis patients are significantly altered compared to controls.

Human fetuin A, also known as α-2-Heremans-Schmid glycoprotein, is a member of the cystatin superfamily of cysteine protease inhibitors. Plasma α-2-HS-glycoprotein is mainly produced by hepatocytes and monocytes/macrophages and is involved in inflammation [35, 36]. Serum α-2-HS-glycoprotein levels are associated with valvular calcification in patients suffering from end-stage renal disease [37]. An inverse association between serum α-2-HS-glycoprotein levels and the prevalence of Aortic Valve Stenosis in non-diabetic patients, but not in diabetic patients without renal disease was shown [38]. In the “label free” proteomic technique, a significant downregulation of α-2-HS-glycoprotein by about 1.4-fold is seen in plasma of Mitral Stenosis patients. Verification studies also reveal significantly diminished plasma α-2-HS-glycoprotein levels in Mitral Stenosis subjects compared to controls. Recently lower serum α-2-HS-glycoprotein levels have been reported in Turkish patients with Rheumatic Mitral Valve disease [39]. This could be because the synthesis of α-2-HS-glycoprotein remains downregulated during injury and inflammation. This may also suggest that α-2-HS-glycoprotein plays a significant role in mitral valve calcification since it acts as an endogenous inhibitor of ectopic calcification. Hence a downregulation of α-2-HS-glycoprotein might lead to improper mineralization by accelerating calcium salt deposition in stenotic mitral valves.

Apolipoprotein AI, a single polypeptide is a major component of HDLs. Several cardioprotective effects of HDL have been attributed to Apo AI. In our verification experiments, apolipoprotein AI levels are significantly lower in Rheumatic Mitral Stenosis subjects compared to controls probably because of decreased protein synthesis, accelerated HDL catabolism and Apo AI replacement by serum amyloid A [40] or the persistence of a chronic inflammation. Apo AI is also well known for its anti-inflammatory properties [41, 42]. Clusterin or apolipoprotein J is a secreted multifunctional protein that was named for its ability to induce cellular clustering. It may function as a chaperone of misfolded extracellular proteins. Apo J has been suggested to have anti-inflammatory [43], cytoprotective [44, 45] and antiapoptotic properties [46]. Experimental evidence suggests that clusterin or Apo J interacts with Apo AI *in vitro* which could be physiologically important [47]. In the present study, plasma clusterin levels differed significantly between the control and patient groups.

Vitronectin (VN), or serum spreading factor and belonging to the group of Arg-Gly-Asp (RGD) type of cell adhesion glycoprotein in plasma, regulates the immune and hemostatic systems particularly at the blood-endothelium interface [48]. Through binding to different integrins, VN may directly interact with endothelial cells and platelets [49–51]. In our study, plasma vitronectin levels are found to be significantly lower in Mitral Stenosis subjects compared to controls. This may imply reduced protection against complement sublytic and lytic attacks as both clusterin and vitronectin are known to regulate the complement pathway by inhibiting complement mediated cell lysis [52]. Very recently, reduced plasma vitronectin concentrations have been observed in Rheumatic Valvular Disease subjects in a Chinese population [53]. In addition, vitronectin plays a role in avian cardiac valve development [54]. Taken together, the findings from the present study indicate that concentration change in vitronectin might be related to valvular pathological changes and may also indicate likely genetic defects in such patients.

The present findings are based on a limited number of representative patient samples. A larger multi-centric population would have been more effective for the verification studies. Again, the present study provided description of the changes in Mitral Stenosis at protein level only. Confounding factors of the disease like age, left atrial diameter, mitral valve area and pulmonary artery systolic pressure may influence alteration of proteins in Mitral Stenosis. Moreover, plasma proteins at high and medium concentrations were mainly detected using this approach. Many other proteins in the ng/ml range like cytokines, small peptides and growth factors were not detected. However, it is believed that the more abundant proteins could play an important role in the development of valvular disorders and as such, their abundances may be altered in pathological circumstances. As expected, we found alterations in quite a few abundant plasma proteins. Lastly, fold change analysis for each protein in equalized plasma samples could not be performed as the CPLL technique usually normalizes the abundances of most proteins. Nevertheless, this is the most comprehensive study performed till date on the plasma proteome in Rheumatic Mitral Stenosis.

## Conclusion

The molecular events leading to disease development are complex and diverse and remain incompletely characterized so far. The identification, quantification and functional characterization of proteins are essential to fully understand these molecular events. Very few proteomic studies on rheumatic valve disease have been carried out till date and none at all on stenotic mitral valve disorder. Our results highlight that Rheumatic Mitral Stenosis is an active inflammatory process which manifests both inflammatory and thrombotic components. Although our findings are purely exploratory, this study provides an example of how one can attempt to identify an entire population of plasma proteins that characterize a disease state. Thus, label free plasma proteomic analysis in Rheumatic Mitral Stenosis for the very first time proves to be a useful strategy to study proteins, some of which might play important roles in the pathophysiology of Mitral Stenosis. Hence, this protein profile may serve as a focal point for future mechanistic studies. Besides some of the proteins found to be differentially abundant in this disorder may be candidate biomarkers for disease diagnosis and prognosis for which validation studies in individual patients in larger populations across multiple centres need to be carried out. These results may help to provide additional information about the molecular mechanisms of Mitral Stenosis and improve the existing diagnostic strategies.

## Methods

### Ethics approval

The study protocol conformed to the principles of the Declaration of Helsinki and was approved by the Institutional Review Boards of the Indian Institute of Chemical Biology, Kolkata and IPGME&R-SSKM, Kolkata, India. Written informed consent was obtained from all subjects prior to their inclusion in the above study.

### Study design and population

The study population consisted of 54 subjects which included 19 controls and 35 chronic Rheumatic Mitral Stenosis patients. Moderate to severe Mitral Stenosis subjects who visited Cardiothoracic and vascular surgery (CTVS) department of the Institute of Post Graduate Medical Education and Research- Seth Sukhlal Karnani Memorial Hospital (IPGME&R-SSKM), Kolkata, India were recruited into the study. Healthy individuals with no apparent cardiac illness or history of coronary artery disease or past history of rheumatic fever or any medication use were selected as controls and underwent the same protocol. All controls had normal blood pressure, cardiac function and lipid profile. They also had normal fasting blood glucose levels.

### Inclusion/Exclusion criteria

Subjects having clinically documented rheumatoid arthritis, diabetes, hypertension, severe systemic illness, renal failure, liver failure or any organic heart disease except Rheumatic Heart Disease were excluded from the study. Even Rheumatic Heart Disease patients presenting with predominantly stenotic or regurgitant lesions of the aortic valve or having predominant regurgitation of the mitral valve were excluded. Other exclusion criteria included malignancies, other inflammatory and connective tissue disorders.

### Echocardiography

Two dimensional transthoracic echocardiography was performed on all subjects using commercially available systems. Echocardiographic diagnosis of Rheumatic Mitral Stenosis was done following the guidelines laid down in the 2012 World Heart Federation criteria [55]. Left atrial and left ventricular diameters both at systole and diastole were assessed. Mitral valve area (MVA) was determined both by planimetry and pressure half time (PHT) methods. Pulmonary artery systolic pressure (PASP) was also measured. Ejection fraction was determined by Simpson’s method. None of the subjects selected for the study had heart failure as assessed by an ejection fraction ≤ 50%.

### Clinical examination

All subjects were examined by physicians and their clinical data entered in a structured pro forma. Symptoms of patients were categorized according to the NYHA functional class. Systolic blood pressure (SBP) and diastolic blood pressure (DBP) were determined. Pulse rate was observed and any irregularity was noted. Latest electrocardiograms (ECGs) were evaluated.

### Sample collection and processing

Peripheral venous blood (~7-8 ml) was drawn aseptically from Mitral Stenosis patients (n = 35) and control subjects (n = 19) during routine clinical assessment (RCA). In the “discovery” or “proteomic analysis” phase, 6 patient samples and 6 control samples were employed for profiling analyses. For better understanding, the demographics, disease severity as well as medication of these 6 selected patients are presented in Tables 2 and 3 respectively and are indicated in italics. The remaining plasma samples were used in the verification experiments. Some of the control and patient samples used in the profiling phase were also included in the verification studies (Additional file 3). Blood samples were collected from controls and from patients prior to mitral valve replacement surgery in sterile (13 × 75 mm × 3 ml) vacutainers (BD, Franklin Lakes, NJ, USA) containing spray dried K2 EDTA as anticoagulant. Plasma was obtained by processing within 3 hours of blood collection. Briefly, the tubes were rolled end to end for about 10 times followed by centrifugation at 2000–2500 × g for 15 mins at 4°C. The upper 2/3 part or the supernatant was collected as plasma and aliquoted in 400-500 μl or less in sterile 1.5 ml microcentrifuge tubes. Samples were labelled and immediately stored in -80°C or liquid nitrogen until further analysis. Repeated freeze-thaw cycles were avoided. Protein concentrations of crude plasma samples were determined using a Lowry assay [56].

### Enrichment of the low abundance plasma proteome on hexapeptide ligand library beads

To decrease sample complexity and to increase the sensitivity of detection we used the CPLL technology. This method is very reproducible, enabling the flow-through fractions (medium to low abundant proteins) to be combined after several elution steps in order to obtain the amounts of protein necessary for high throughput proteomic analysis. Plasma samples collected from healthy individuals (n = 3) and stenotic patients (n = 3) were pooled and pre-treated with the ProteoMiner protein enrichment kit (Bio-Rad Laboratories, Hercules, CA, USA) according to the manufacturer’s instructions. The eluted fractions from the patient and control spin columns were pooled and stored at -20°C prior to analysis.

### Protein precipitation, quantification and in-solution trypsin digestion

Proteins obtained by the enrichment method were precipitated in four sample volumes of ice-cold acetone (100%) overnight at -20°C. Samples were then centrifuged at 14000 × g for 10 min and pellets were dissolved in 100 μl of 20 mM NH_4_HCO_3_. Protein concentration was determined by DC protein assay (Bio Rad, Hercules, CA, USA) according to the manufacturer’s instructions. For in-solution digestion, proteins (100 μg) from crude and equalized plasmas were solubilized with 0.1% RapiGest SF (Waters Corporation, Milford, MA,USA) in 50 mM NH_4_HCO_3_ to enhance proteolytic cleavage. Proteins were reduced and alkylated with 100 mM DTT at 37°C for 45–60 min and 200 mM iodoacetamide in the dark at room temperature for 45–60 min, respectively. The proteins were digested overnight (~16-18 hrs) with sequencing grade porcine trypsin (Promega, Madison, WI, USA) in 50:1 ratio at 37°C. The reaction was stopped by incubating with 2 μl of 50% formic acid at 37°C for 20 minutes. The digest was vortexed and centrifuged to collect the supernatant containing peptides.

### LC-MS^E^

LC-MS^E^, a label free mass spectrometry based shotgun proteomics technique was adopted for quantification of differentially abundant proteins as described earlier [57].

#### UPLC conditions

400 ng of digested peptides after reconstituting in 3% acetonitrile, 0.1% formic acid were injected into online nanoACQUITY UPLC coupled to a QTOF, Synapt-HDMS mass spectrometer (Waters Corporation, Milford, MA, USA). The peptides were separated by using a BEH 130-C_18_ reversed phase column (1.7 μm x 75 μm x 250 mm) (Waters Corporation, Milford, MA, USA). The binary solvent system used comprised 99.9% water and 0.1% formic acid (mobile phase A) and 99.9% acetonitrile and 0.1% formic acid (mobile phase B). Peptides were initially preconcentrated and desalted online at a flow rate of 5 μl/min using a 5 μm Symmetry C_18_ trapping column (internal diameter 180 mm, length 20 mm) (Waters Corporation, Milford, MA,USA) with a 0.1% B. After each injection, peptides were eluted into the NanoLockSpray ion source at a flow rate of 300 nL/min using a gradient of 2– 40% B for 90 min. Then the column was washed and equilibrated.

#### Mass spectrometry conditions

Q-TOF MS/MS was performed at a resolution of about 10000 full width half maximum (FWHM) using an ESI interface. For instrument calibration, the external calibration standard or lock mass [(GLU1)]-Fibrinopeptide B,50 fmol/μl) was constantly infused by the NanoACQUITY auxiliary pump at a constant flow rate of 500nL/min at an interval of 20 secs (lock spray frequency) and the mass error was less than 2 ppm. The lock mass data were averaged for correction. The eluted peptide spectra were acquired by Synapt HDMS (Q-TOF) in a positive V mode in a scan mass range of 50–2000 m/z with a scan time of 0.7 sec. The on-line eluted peptides were analyzed at both low collision energy (4 eV) and high collision energy (15-40 eV). The tryptic digests generated either from crude or equalized plasma, were run in triplicates.

#### Protein identification

LC-MS^E^ data analysis was performed with the Protein Lynx Global Server 2.5.2 software (PLGS; Waters Corporation, Milford, MA, USA). Preliminary searches were made for protein identification against reviewed *Homo sapiens* database (UniProtKB) using the search engine Integrated protein lynx global server [58]. Precursor and peptide fragment mass tolerance was selected “automatic” in the PLGS workflow. PLGS identifies proteins with less than 10–15 ppm for precursor and products (50 ppm). Oxidation of methionine and carbamidomethylation of cysteine were searched as variable and fixed modifications, enzyme specificity was trypsin and upto two missed cleavages were allowed. The false positive rate was kept at 4% in the PLGS workflow. The theoretical molecular mass (MW) and isoelectric point (pI) were determined for each of the identified proteins using ExPASy Bioinformatics Resource Portal (http://www.expasy.org/compute_pi/). Only those proteins were considered identified where a same protein was identified by PLGS in all three technical replicates or in at least two of the three runs. Proteins showing a trend of upregulation in the proteomic analysis are shown in Additional file 4.

#### Label free protein quantification

Label free protein quantification was done by MS^E^ approach. As an internal detection control, 100 fmol of a digest of yeast alcohol dehydrogenase [Swiss-Prot: P00330] was spiked with the protein digest for quantification. Three most intense peptides of the spiked protein were considered for quantification. Data was acquired in three technical replicates and out of those the one which showed highest nanogram concentration was used for normalization of control and patient runs respectively. Accurate mass and retention time matches of precursors were compared across all LC-MS runs for label-free intensity based quantitation. The ratio or fold change for each altered protein as also the protein ratio p values for differential abundance were computed by PLGS.

### Functional annotation of profiled proteins

Major functions of overlapping and differentially abundant proteins were determined using the Swiss- Prot database [59].

### Verification of selected proteins by quantitative ELISA

Plasma concentrations of three selected proteins- α-2-HS- glycoprotein (R&D Systems, Minneapolis, MN, USA), clusterin (R&D Systems, Minneapolis, MN, USA) and vitronectin (Takara Bio Inc. Shiga, Japan), were determined in individual patient and control plasma by enzyme immunoassay (EIA) using commercial assay systems following the manufacturer’s protocol. Duplicate measurements were taken and averaged. Plasma α-2-HS- glycoprotein was assessed in 10 controls and 18 patient subjects while clusterin was measured in 10 controls and 15 diseased subjects. Plasma vitronectin was measured in 10 controls and 14 patients. Plasma apolipoprotein AI concentrations were quantitatively assessed in 12 control and 13 patient plasma samples using an immunoturbidimetric assay (Randox Laboratories). No detectable cross reaction or interference was observed for any of the four proteins assayed. The minimum detection sensitivities ranged from 0.16-1.74 ng/ml for α-2-HS- glycoprotein and 0.06-1.05 ng/ml for clusterin while for vitronectin it was 5 ng/ml. The inter and intra-assay variations ranged between 2.2-9.6% and 4.9-6.3% for vitronectin, 6.8-8.4% and 3.4-3.7% for clusterin and 7.3-8.4% and 3.9-4.9% for α-2-HS-glycoprotein respectively.

### Identification of pathways associated with rheumatic mitral stenosis

In order to better understand the relevance of the identified proteins in the disease context, pathway analysis was done using the Database for Annotation, Visualization and Integrated Discovery (DAVID) bioinformatics resources (david.abcc.ncifcrf.gov/tools.jsp). For this, UniProt human accession numbers were uploaded as a “Gene List” with Identifier selected as “UNIPROT_ACCESSION”. Finally, the DAVID tool [60] was used as an interface to the KEGG [61] pathway repository at Kyoto University to generate pathway diagrams that illustrate some possible association of the common and altered proteins with the pathophysiology of Rheumatic Mitral Stenosis.

### Histopathology analysis

Anterior leaflets of mitral valve tissue samples, from subjects undergoing valve replacement and from post mortem control subjects without any known history of cardiovascular disease, were collected and fixed in 10% formalin. Tissue samples were then embedded in paraffin and 5 μm thick sections were prepared. Sections were then stained with HE stain (Sigma Chemical Co., St Louis, MO, USA) as described earlier [62]. Sections were examined under an Olympus BX51 (Olympus Corporation, Tokyo, Japan) microscope and images were captured with a digital camera attached to it.

### Analysis of protein interaction networks

Protein interaction networks for the differentially abundant as well as the common proteins were computed by STRING version 9.1 (http://string-db.org/). STRING nodes are proteins and edges are the predicted functional associations based on primary databases such as KEGG and GO, and primary literature. STRING predicts these interactions based on neighbourhood, gene fusion products, homology and similarity of coexpression patterning. Network interaction scores for each node are expressed as a joint probability derived from curated databases of experimental information, text mining and computationally predicted by genetic proximity [63]. In this study, STRING networks were calculated with the following default settings - medium confidence score: 0.400, network depth: 0 and up to 50 interactions.

### Statistical analysis

Data are expressed as mean ± standard error of mean (SEM). Categorical variables were analysed by Chi square (*χ*^2^) or Fisher’s exact test. Differences between the control and patient groups were tested by a Student’s t- test for unpaired data once normality was demonstrated by Shapiro Wilk test. Venn diagram was done with Microsoft excel 2010. A probability value < 0.05 was considered statistically significant.

## Electronic supplementary material

Additional file 1: **List of proteins identified in plasma samples of controls.** (PDF 202 KB)

Additional file 2: **List of proteins identified in plasma samples of Rheumatic Mitral Stenosis.** (PDF 209 KB)

Additional file 3: **Details of profiling and verification samples.** (PDF 68 KB)

Additional file 4: **List of proteins showing a trend of upregulation in the proteomic analysis.** (PDF 13 KB)

## Abbreviations

ACE: Angiotensin converting enzyme
ESI: Electrospray ionization
GO: Gene ontology
KEGG: Kyoto encyclopedia of genes and genomes
LC-MS^E^: Liquid chromatography mass spectrometry at elevated energy
QTOF: Quadrupole time of flight
UPLC: Ultra performance liquid chromatography.
