# Supplementary material for: Proteomic analysis of human plasma in chronic rheumatic mitral stenosis reveals proteins involved in the complement and coagulation cascade
Source: Clin Proteomics. 2014 Sep 24;11(1):35. doi: 10.1186/1559-0275-11-35 (PMC4193131; doi:10.1186/1559-0275-11-35)
Supplement: Supplementary file 1 — Additional file 1: List of proteins identified in plasma samples of controls. (PDF 202 KB) [file 12014_2014_78_MOESM1_ESM.pdf]

List of proteins identified in plasma samples of controls. CPLL, Combinatorial Peptide Ligand Library; MW, Experimental Molecular Weight; pI Experimental Isoelectric point; RP, Raw Plasma; *Th* MW, Theoretical Molecular Weight; *Th* pI Theoretical Isoelectric point.

Accession No.	Description	MW (Da)	Th MW(Da)	pI	Th pI	Seq. Coverage in RP(%)	Seq. Coverage in CPLL(%)
P01857	Ig gamma 1 chain C region	36083	36083	8.18	8.46	82	
P01834	Ig kappa chain C region	11601	11601	5.50	5.58	85	36
P01859	Ig gamma 2 chain C region	35877	35877	7.44	7.66	71	
P01876	Ig alpha 1 chain C region	37630	37630	6.06	6.08	60	5.0
P01860	Ig gamma 3 chain C region (HDC) (Heavy chain disease protein)	41260	41260	7.79	8.23	61	
P01871	Ig mu chain C region	49275	49275	6.33	6.35	39	31
P01861	Ig gamma 4 chain C region	35917	35917	7.11	7.18	54	
P01024	Complement C3	187029	187029	5.96	6.02	55	
P02763	Alpha 1 acid glycoprotein 1	23496	23496	4.74	4.93	38	
P01765	Ig heavy chain V III region TIL	12348	12348	9.41	9.23	20	
P01877	Ig alpha 2 chain C region	36503	36503	5.66	5.71	53	
P01620	Ig kappa chain V III region SIE	11767	11767	8.79	8.7	72	
P01625	Ig kappa chain V IV region Len	12632	12632	8.17	7.92	21	
P0C0L5	Complement C4 B	192671	192631	6.72	6.89	36	41
P04004	Vitronectin	54271	54271	5.43	5.55	40	19

P01596	Ig kappa chain V I region CAR	11696	11696	9.72	9.47	17	
P19652	Alpha 1 acid glycoprotein 2	23587	23587	4.85	5.03	31	
P01766	Ig heavy chain V III region BRO	13218	13218	6.50	6.44	38	18
P08603	Complement factor H	139004	139004	6.18	6.21	42	11
P00751	Complement factor B (EC 3.4.21.47)	85478	85478	6.66	6.67	21	
P0C0L4	Complement C4 A	192649	192665	6.64	6.66	39	
P04003	C4b binding protein alpha chain	66989	66989	6.99	7.15	31	14
P01781	Ig heavy chain V III region GAL	12722	12722	8.81	8.7	33	
O43866	CD5 antigen like	38062	38062	5.14	5.28	34	
Q96PD5	N acetylmuramoyl L alanine amidase (EC 3.5.1.28)	62177	62177	7.27	7.25	17	
P01605	Ig kappa chain V I region Lay	11826	11826	8.20	7.96	68	
P23083	Ig heavy chain V I region V35	13000	13000	9.79	9.59	23	
P01593	Ig kappa chain V I region AG	11984	11984	5.57	5.57	58	
P04220	Ig mu heavy chain disease protein	43030	43030	4.95	5.13		23
P35542	Serum amyloid A 4 protein	14737	14737	9.38	9.17		26
P02747	Complement C1q subcomponent subunit C	25757	25757	8.54	8.61		14
P09871	Complement C1s subcomponent (EC 3.4.21.42)	76634	76634	4.66	4.85		15
P18428	Lipopolysaccharide binding protein	53350	53350	6.24	6.23		8

P0CG05	Ig lambda 2 chain C regions	11286	11286	7.07	6.91	76	46
P01777	Ig heavy chain V III region TEI	12794	12794	8.79	8.72	16	
P04433	Ig kappa chain V III region VG Fragment	12567	12567	4.65	4.85	8	
P01009-2	Isoform 2 of Alpha 1 antitrypsin	40237	40731	5.12	5.20	46	
P01611	Ig kappa chain V I region Wes	11600	11600	7.16	6.91	17	
P01774	Ig heavy chain V III region POM	12945	12945	8.20	8.05	20	
P18136	Ig kappa chain V III region HIC	14080	14080	6.56	6.19	50	
P01871-2	Isoform 2 of Ig mu chain C region	51757	51757	5.73	5.80	35	
P01596	Ig kappa chain V I region CAR	11696	11696	9.72	9.47	25	
P01700	Ig lambda chain V I region HA	11888	11888	9.21	9.07	12	
P0CG06	Ig lambda 3 chain C regions	11230	11230	7.07	6.91	74	
P0CG04	Ig lambda 1 chain C regions	11340	11340	7.99	7.89	32	
P01623	Ig kappa chain V III region WOL	11738	11738	9.21	9.07	21	
P04208	Ig lambda chain V I region WAH	11717	11717	6.4438	6.29	12	
P80748	Ig lambda chain V III region LOI	11927	11927	4.7563	4.94	29	
P02768	Serum albumin	69321	69321	5.86	5.92	79	20
P02787	Serotransferrin	77013	77013	6.75	6.81	65	
P02790	Hemopexin	51643	51643	6.57	6.55	52	
P68871	Hemoglobin subunit beta	15988	15988	6.88	6.74	86	
P69905	Hemoglobin subunit alpha	15247	15247	9.18	8.72	62	
P02774	Vitamin D binding protein	52929	52929	5.24	5.4	53	
P02774-2	Isoform 2 of Vitamin D binding protein	39516	39516	4.98	5.16	42	

P00450	Ceruloplasmin (EC 1.16.3.1)	12212 7	122127	5.34	5.44	43	14
P02766	Transthyretin	15877	15877	5.39	5.39	49	28
P02042	Hemoglobin subunit delta	16045	16045	8.24	7.84	54	
P00738	Haptoglobin	45176	45177	6.12	6.13	49	
P01023	Alpha 2 macroglobulin	16318 7	163187	6.00	6.03	46	
P01009	Alpha 1 antitrypsin	46707	46707	5.24	5.37	47	
P01009-3	Isoform 3 of Alpha 1 antitrypsin	34733	34733	4.87	5.04	41	
P02765	Alpha 2 HS glycoprotein	39299	39299	5.33	5.43	30	13
P01011	Alpha 1 antichymotrypsin	47620	47620	5.18	5.33	25	
P04196	Histidine rich glycoprotein	59540	59540	7.10	7.09	23	
P25311	Zinc alpha 2 glycoprotein	34237	34237	5.64	5.71	27	
P19827	Inter alpha trypsin inhibitor heavy chain H1	10132 5	101325	6.31	6.31	20	
P19823	Inter alpha trypsin inhibitor heavy chain H2	10639 6	106396	6.40	6.4	20	10
P02760	Protein AMBP	38973	38973	5.87	5.87	34	
P08697	Alpha 2 antiplasmin	54531	54531	5.84	5.87	14	
P08697-2	Isoform 2 of Alpha 2 antiplasmin	47876	47876	5.67	5.73	23	
P01019	Angiotensinogen	53120	53120	5.85	5.87	24	
Q14624	Inter alpha trypsin inhibitor heavy chain H4	10329 3	103293	6.53	6.51	15	
Q14624-2	Isoform 2 of Inter alpha trypsin inhibitor heavy chain H4	10117 7	101177	6.20	6.21	22	
P05155	Plasma protease C1 inhibitor	55119	55119	6.08	6.09	23	

P22792	Carboxypeptidase N subunit 2	60518	60518	5.57	5.63	11	
P02679-2	Isoform Gamma A of Fibrinogen gamma chain	49464	49464	5.64	5.7	46	
P02671-2	Isoform 2 of Fibrinogen alpha chain	69713	69713	8.01	8.23	27	
P02749	Beta 2 glycoprotein 1	38272	38272	7.87	8.34	48	
P02675	Fibrinogen beta chain	55892	55892	8.25	8.54	55	47
P01042	Kininogen 1	71912	71912	6.34	6.34	19	
P01042-2	Isoform LMW of Kininogen 1	47852	47852	6.26	6.29	36	
P01042-3	Isoform 3 of Kininogen 1	43793	43793	6.02	6.07	19	
P01008	Antithrombin III	52568	52568	6.30	6.32	37	
P00734	Prothrombin (EC 3.4.21.5)	69992	69992	5.53	5.63	37	17
P00747	Plasminogen (EC 3.4.21.7)	90510	90510	6.91	7.04	31	
P02679	Fibrinogen gamma chain	51478	51478	5.24	5.37	42	42
P02671	Fibrinogen alpha chain	94914	94914	5.61	5.7	21	31
P07225	Vitamin K dependent protein S	75074	75074	5.34	5.48		5
P02647	Apolipoprotein A I	30758	30758	5.43	5.56	61	71
P02652	Apolipoprotein A II	11167	11167	6.64	6.27	51	30
P10909-2	Isoform 2 of Clusterin	57795	57795	6.23	6.24	22	
P10909-3	Isoform 3 of Clusterin	32342	32342	6.19	6.19	22	
P10909-4	Isoform 4 of Clusterin (Apolipoprotein J)	48772	49142	6.26	6.17	25	
P02656	Apolipoprotein C III	10845	10845	5.06	5.23	45	53
P06727	Apolipoprotein A IV	45371	45371	5.11	5.28	36	38
P05090	Apolipoprotein D	21261	21261	4.87	5.06	28	12

P02649	Apolipoprotein E	36131	36131	5.48	5.65	25	56
P02654	Apolipoprotein C I	9326	9326	9.40	8.01		27
P10909	Clusterin	52461	52461	5.84	5.88		29
P02655	Apolipoprotein C II	11276	11276	4.44	4.64	35	20
P27169	Serum paraoxonasearylesterase 1 (EC 3.1.1.2) (EC 3.1.1.81) (EC 3.1.8.1) (PON 1)	39706	39706	4.92	5.08	18	33
P04217	Alpha 1B glycoprotein	54219	54219	5.48	5.56	31	
Q6X4W1-4	Isoform 4 of Nasal embryonic luteinizing hormone releasing hormone factor	56568	56568	9.33	9.2	23	
P00739	Haptoglobin related protein	39004	39004	6.67	6.63	40	
P20742	Pregnancy zone protein	163759	163759	5.9326	5.97	7	
