# Supplementary material for: Proteomic analysis of human plasma in chronic rheumatic mitral stenosis reveals proteins involved in the complement and coagulation cascade
Source: Clin Proteomics. 2014 Sep 24;11(1):35. doi: 10.1186/1559-0275-11-35 (PMC4193131; doi:10.1186/1559-0275-11-35)
Supplement: Supplementary file 2 — Additional file 2: List of proteins identified in plasma samples of Rheumatic Mitral Stenosis. (PDF 209 KB) [file 12014_2014_78_MOESM2_ESM.pdf]

List of proteins identified in plasma samples of Rheumatic Mitral Stenosis. CPLL, Combinatorial Peptide Ligand Library; MW, Experimental Molecular Weight; pI Experimental Isoelectric point; RP, Raw Plasma; *Th* MW, Theoretical Molecular Weight; *Th* pI Theoretical Isoelectric point.

Accession No.	Description	MW (Da)	Th MW(Da)	pI	Th pI	Seq. Coverage in RP(%)	Seq. Coverage in CPLL(%)
P01857	Ig gamma 1 chain C region	36083	36083	8.18	8.46	62	26
P01834	Ig kappa chain C region	11601	11601	5.50	5.58	78	53
P01859	Ig gamma 2 chain C region	35877	35877	7.44	7.66	49	
P01876	Ig alpha 1 chain C region	37630	37630	6.06	6.08	57	17
P01860	Ig gamma 3 chain C region	41260	41260	7.79	8.23	48	
P01871	Ig mu chain C region	49275	49275	6.33	6.35	41	32
P01861	Ig gamma 4 chain C region	35917	35917	7.11	7.18	64	
P0CG04	Ig lambda 1 chain C regions	11340	11340	7.99	7.89	71	
P01024	Complement C3	187029	187029	5.96	6.02	54	18
P02763	Alpha 1 acid glycoprotein 1	23496	23496	4.74	4.93	43	
P01765	Ig heavy chain V III region TIL	12348	12348	9.41	9.23	28	
P01877	Ig alpha 2 chain C region	36503	36503	5.66	5.71	54	19
P01620	Ig kappa chain V III region SIE	11767	11767	8.79	8.7	59	
P01625	Ig kappa chain V IV region Len	12632	12632	8.17	7.92	32	
P0C0L5	Complement C4 B	192671	192631	6.72	6.89	35	43

P04004	Vitronectin	54271	54271	5.43	5.55	21	20
P19652	Alpha 1 acid glycoprotein 2	23587	23587	4.85	5.03	40	
P01766	Ig heavy chain V III region BRO	13218	13218	6.50	6.44	37	
P08603	Complement factor H	139004	139004	6.18	6.21	36	11
P00751	Complement factor B (EC 3.4.21.47)	85478	85478	6.66	6.67	33	
P0C0L4	Complement C4 A	192649	192664	6.64	6.66	37	
P04003	C4b binding protein alpha chain)	66989	66989	7.00	7.15	21	13
Q96PD5	N acetylmuramoyl L alanine amidase (EC 3.5.1.28)	62177	62177	7.27	7.25	27	
P35542	Serum amyloid A 4 protein	14737	14737	9.38	9.17	21	
O43866	CD5 antigen like	38062	38062	5.14	5.28	20	
P02747	Complement C1q subcomponent subunit C	25757	25757	8.54	8.61		29
P09871	Complement C1s subcomponent (EC 3.4.21.42)	76634	76634	4.66	4.85		23
P18428	Lipopolysaccharide binding protein)	53350	53350	6.24	6.23		12
P0CG05	Ig lambda 2 chain C regions	11286	11286	7.07	6.91	77	43
P0CG06	Ig lambda 3 chain C regions	11230	11230	6.91	7.07	77	
P01617	Ig kappa chain V II region TEW	12308	12308	5.69	5.59	33	
P01598	Ig kappa chain V I region EU	11780	11780	8.62	8.78	29	
P02748	Complement component C9	63132	63132	5.43	5.27	24	

P02743	Serum amyloid P component	25371	25371	6.1	6.10	27	
P02735	Serum amyloid A protein	13523	13523	6.35	6.28	34	
P0DJI8	Serum amyloid A 1 protein	13523	13523	6.35	6.28	31	
P02746	Complement C1q subcomponent subunit B	26704	26704	8.83	8.85		21
Q03591	Complement factor H related protein 1	37625	37625	7.39	7.19		14
P01591	Immunoglobulin J chain	18086	18086	5.09	4.91	13	11
Q08380	Galectin 3 binding protein	65289	65289	5.12	4.94		16
O75636	Ficolin 3	32881	32881	6.2	6.21		17
P01871-2	Isoform 2 of Ig mu chain C region	51757	51757	5.73	5.80	38	
P01777	Ig heavy chain V III region TEI	12794	12794	8.79	8.72	23	
P01593	Ig kappa chain V I region AG	11984	11984	5.57	5.67	31	
P01764	Ig heavy chain V III region VH26	12574	12574	8.46	8.49	28	
P01774	Ig heavy chain V III region POM	12945	12945	8.20	8.05	12	
P01700	Ig lambda chain V I region HA	11888	11888	9.21	9.07	12	
P01596	Ig kappa chain V I region CAR	11696	11696	9.72	9.47	29	
P02768	Serum albumin	69321	69321	5.86	5.92	81	29
P02787	Serotransferrin	77013	77013	6.75	6.81	66	
P02790	Hemopexin	51643	51643	6.57	6.55	48	4
P68871	Hemoglobin subunit beta	15988	15988	6.88	6.74	56	
P69905	Hemoglobin subunit alpha	15247	15247	9.18	8.72	58	
P02774	Vitamin D binding protein	52929	52929	5.24	5.4	47	
P00450	Ceruloplasmin (EC 1.16.3.1)	122127	122127	5.34	5.44	38	33

P02766	Transthyretin	15877	15877	5.40	5.40	44	54
P02768-2	Isoform 2 of Serum albumin	47329	47329	5.97	5.92	80	
P43652	Afamin	69024	69024	5.64	5.54	20	
P04278	Sex hormone binding globulin	43751	43751	6.22	6.24		15
P00738	Haptoglobin	45176	45176	6.12	6.13	59	
P01023	Alpha 2 macroglobulin	163187	163187	6.00	6.03	47	
P01009	Alpha 1 antitrypsin	46707	46707	5.24	5.37	53	25
P02765	Alpha 2 HS glycoprotein	39299	39299	5.33	5.43	30	11
P01011	Alpha 1 antichymotrypsin	47620	47620	5.18	5.33	33	
P04196	Histidine rich glycoprotein	59540	59540	7.10	7.09	17	
P19827	Inter alpha trypsin inhibitor heavy chain H1	101325	101325	6.31	6.31	16	
P19823	Inter alpha trypsin inhibitor heavy chain H2	106396	106396	6.40	6.4	19	13
P02760	Protein AMBP	38973	38973	5.87	5.87	21	
P01019	Angiotensinogen	53120	53120	5.85	5.87	19	
P05546	Heparin cofactor 2	57034	57034	6.41	6.43	9	
Q14624	Inter alpha trypsin inhibitor heavy chain H4	103293	103293	6.51	6.53	20	15
Q14624-2	Isoform 2 of Inter alpha trypsin inhibitor heavy chain H4	101177	101177	6.20	6.21	21	
P05155	Plasma protease C1 inhibitor	55119	55119	6.08	6.09	19	
P02679-2	Isoform Gamma A of Fibrinogen gamma chain	49464	49464	5.64	5.7	46	

P02671-2	Isoform 2 of Fibrinogen alpha chain	69713	69713	8.01	8.23	38	
P02749	Beta 2 glycoprotein 1	38272	38272	7.87	8.34	38	
P02675	Fibrinogen beta chain	55892	55892	8.25	8.54	64	56
P01042	Kininogen 1	71912	71912	6.34	6.34	18	
P01042-2	Isoform LMW of Kininogen 1	47852	47852	6.26	6.29	24	
P01008	Antithrombin III	52568	52568	6.30	6.32	30	
P00734	Prothrombin (EC 3.4.21.5)	69992	69992	5.53	5.63	31	17
P00747	Plasminogen (EC 3.4.21.7)	90510	90510	6.91	7.04	37	
P02679	Fibrinogen gamma chain	51478	51478	5.24	5.37	43	48
P02671	Fibrinogen alpha chain	94914	94914	5.61	5.7	28	33
P07225	Vitamin K dependent protein S	75074	75074	5.34	5.48		16
P02647	Apolipoprotein A I	30758	30758	5.43	5.56	61	74
P02652	Apolipoprotein A II	11167	11167	6.64	6.27	38	43
P10909-4	Isoform 4 of Clusterin	48772	49142	6.26	6.17	24	
P02656	Apolipoprotein C III	10845	10845	5.06	5.23	42	51
P06727	Apolipoprotein A IV	45371	45371	5.11	5.28	39	61
P05090	Apolipoprotein D	21261	21261	4.87	5.06	17	
P02649	Apolipoprotein E	36131	36131	5.48	5.65	31	58
P02654	Apolipoprotein C I	9326	9326	9.40	8.01	24	28
P10909	Clusterin	52461	52461	5.84	5.88	19	33
P02655	Apolipoprotein C II	11276	11276	4.44	4.64		39

P25311	Zinc alpha 2 glycoprotein	34237	34237	5.71	5.64	34	
P80108	Phosphatidylinositol glycan specific phospholipase D (EC 3.1.4.50)	92278	92278	5.91	5.89		8
P27169	Serum paraoxonase arylesterase 1 (EC 3.1.1.2) (EC 3.1.1.81) (EC 3.1.8.1)	39706	39706	4.92	5.08		47
P22352	Glutathione peroxidase 3 (EC 1.11.1.9)	25385	25536	8.26	8.22		14
P23142	Fibulin 1	77162	77162	5.07	4.90		16
P06396	Gelsolin	85644	85644	5.84	5.9		18
P06396-2	Isoform 2 of Gelsolin	80590	80590	5.58	5.47	16	
P04217	Alpha 1B glycoprotein	54219	54219	5.48	5.56	31	
P04217-2	Isoform 2 of Alpha 1B glycoprotein	40692	40692	5.40	5.50	26	
P20742	Pregnancy zone protein	163759	163759	5.93	5.97	10	
P00739	Haptoglobin related protein	39004	39004	6.67	6.63	46	
