# Supplementary material for: Proteomic analysis of human plasma in chronic rheumatic mitral stenosis reveals proteins involved in the complement and coagulation cascade
Source: Clin Proteomics. 2014 Sep 24;11(1):35. doi: 10.1186/1559-0275-11-35 (PMC4193131; doi:10.1186/1559-0275-11-35)
Supplement: Supplementary file 3 — Additional file 3: Details of profiling and verification samples. (PDF 68 KB) [file 12014_2014_78_MOESM3_ESM.pdf]

**Details of Profiling and Verification samples.** C, control; CPLL, combinatorial peptide ligand library; F, female; M , male; P, patient; RP, raw plasma; Y ,yes

| | | | **PROFILING** | | | | **VALIDATION** | |
|---|---|---|---|---|---|---|---|---|
| | | | | | | | | |
| Number | Age | Sex | RP | CPLL | Fetuin A | Clusterin | ApolipoproteinAI | Vitronectin |
| | | | | | | | | |
| C1 | 45 | F | | | Y | Y | Y | |
| C2 | 28 | F | | | Y | Y | Y | Y |
| C3 | 32 | M | | | Y | Y | Y | |
| C4 | 33 | F | | Y | Y | Y | Y | |
| C5 | 26 | F | | Y | Y | Y | Y | |
| C6 | 22 | M | | | Y | | | |
| C7 | 45 | F | | Y | | Y | Y | |
| C8 | 25 | M | | | Y | | Y | |
| C9 | 36 | M | Y | | Y | Y | | Y |
| C10 | 36 | M | | | Y | | | Y |
| C11 | 46 | M | Y | | | | Y | Y |
| C12 | 24 | F | | | | | Y | |
| C13 | 42 | M | Y | | | | Y | Y |
| C14 | 28 | M | | | | | Y | Y |
| C15 | 26 | M | | | Y | Y | Y | Y |
| C16 | 26 | M | | | | | | |
| C17 | 38 | F | | | | Y | | Y |
| C18 | 28 | F | | | | | | Y |
| C19 | 35 | M | | | | Y | | Y |
| P1 | 41 | M | Y | | | | | Y |
| P2 | 42 | F | | | | Y | | |
| P3 | 45 | M | | | Y | | | |
| P4 | 35 | M | | | Y | | Y | |
| P5 | 24 | M | | | | Y | | |
| P6 | 26 | M | | | Y | | | |
| P7 | 33 | F | | Y | | | Y | |
| P8 | 35 | M | | | | | | Y |
| P9 | 45 | F | | Y | | Y | Y | |
| P10 | 35 | M | | | Y | | | |
| P11 | 33 | M | | | Y | | | |
| | | | | | | | | |

| | | | PROFILING | | | | VALIDATION | |
|---|---|---|---|---|---|---|---|---|
| | | | | | | | | |
| Number | Age | Sex | RP | CPLL | Fetuin A | Clusterin | ApolipoproteinA1 | Vitronectin |
| | | | | | | | | |
| P12 | 36 | F | | | | | Y | Y |
| P13 | 48 | M | | | | | Y | Y |
| P14 | 25 | F | | | | | | Y |
| P15 | 35 | F | | | | | | Y |
| P16 | 32 | F | | | | | | Y |
| P17 | 55 | M | | | Y | Y | | |
| P18 | 33 | F | | | | Y | | |
| P19 | 40 | F | | Y | Y | Y | | |
| P20 | 19 | M | | | | Y | | Y |
| P21 | 38 | F | | | | | | Y |
| P22 | 42 | M | Y | | Y | | | Y |
| P23 | 24 | F | | | Y | | | Y |
| P24 | 27 | M | Y | | Y | | Y | Y |
| P25 | 48 | M | | | Y | Y | Y | |
| P26 | 40 | F | | | Y | Y | | |
| P27 | 28 | M | | | Y | Y | Y | |
| P28 | 35 | F | | | Y | Y | | |
| P29 | 43 | F | | | Y | Y | Y | |
| P30 | 35 | M | | | Y | Y | Y | |
| P31 | 50 | M | | | Y | Y | Y | |
| P32 | 47 | F | | | | | | Y |
| P33 | 46 | M | | | Y | Y | Y | |
| P34 | 30 | M | | | | | | Y |
| P35 | 36 | M | | | | | Y | |
