# Supplementary material for: Proteomic analysis of human plasma in chronic rheumatic mitral stenosis reveals proteins involved in the complement and coagulation cascade
Source: Clin Proteomics. 2014 Sep 24;11(1):35. doi: 10.1186/1559-0275-11-35 (PMC4193131; doi:10.1186/1559-0275-11-35)
Supplement: Supplementary file 4 — Additional file 4: List of proteins showing a trend of upregulation in the proteomic analysis. (PDF 13 KB) [file 12014_2014_78_MOESM4_ESM.pdf]

List of proteins showing a trend of upregulation in the proteomic analysis. Three nano LC-MS^E analyses of the plasma samples were performed for protein identification and relative quantification. The difference in abundances of these proteins were not found to be significant between control and patients except for Apolipoprotein A II and Alpha 1 acid glycoprotein II but these 2 proteins were not consistently altered in triplicate analyses.

Swiss-Prot Accession No	Protein Name	Ratio[Patient/ Control]	p value
P01008	Antithrombin III	1.1	0.16
P02652	Apolipoprotein A II	1.09	0.04
P19652	Alpha 1 acid glycoprotein II	1.82	0.001
P02774	Vitamin D binding protein	1.2	0.7
P02787	Serotransferrin	1.6	1
P02790	Hemopexin	2.15	1
P01042-2	Isoform LMW of Kininogen	3.0	0.98
P69905	Hemoglobin subunit alpha	1.7	1
P68871	Hemoglobin subunit beta	3.0	1
